# Characterization of Angiogenic, Matrix Remodeling, and Antimicrobial Factors in Preterm and Full-Term Human Umbilical Cords

**DOI:** 10.3390/jdb12020013

**Published:** 2024-05-01

**Authors:** Kaiva Zile Zarina, Mara Pilmane

**Affiliations:** Institute of Anatomy and Anthropology, Riga Stradins University, Kronvalda Boulevard 9, LV-1010 Riga, Latvia; zarina.zile@gmail.com

**Keywords:** MMP2, TIMP2, VEGF, CD34, HBD2, umbilical cord

## Abstract

Background: Little is known about morphogenetic changes in the umbilical cord during the maturation process. Extracellular matrix remodeling, angiogenesis, progenitor activity, and immunomodulation are represented by specific markers; therefore, the aim of this study was to determine the expression of matrix metalloproteinase-2 (MMP2), tissue inhibitor of metalloproteinases-2 (TIMP2), CD34, vascular endothelial growth factor (VEGF), and human β-defensin 2 (HBD2) in preterm and full-term human umbilical cord tissue. Methods: Samples of umbilical cord tissue were obtained from 17 patients and divided into two groups: very preterm and moderate preterm birth umbilical cords; late preterm birth and full-term birth umbilical cords. Routine histology examination was conducted. Marker-positive cells were detected using the immunohistochemistry method. The number of positive structures was counted semi-quantitatively using microscopy. Statistical analysis was carried out using the SPSS Statistics 29 program. Results: Extraembryonic mesenchyme cells are the most active cell producers, expressing MMP2, TIMP2, VEGF, and HBD2 at notable levels in preterm and full-term umbilical cord tissue. Statistically significant differences in the expression of CD34, MMP2, and TIMP2 between the two patient groups were found. The expression of VEGF was similar in both patient groups, with the highest number of VEGF-positive cells seen in the extraembryonic mesenchyme. The expression of HBD2 was the highest in the extraembryonic mesenchyme and the amniotic epithelium, where mostly moderate numbers of HBD2-positive cells were detected. Conclusions: Extracellular matrix remodeling in preterm and term umbilical cords is strongly regulated, and tissue factors MMP2 and TIMP2 take part in this process. The expression of VEGF is not affected by the umbilical cord’s age; however, individual patient factors can affect the production of VEGF. Numerous CD34-positive cells in the endothelium of the umbilical arteries suggest a significant role of progenitor cells in very preterm and moderate preterm birth umbilical cords. Antimicrobial activity provided by HBD2 is essential and constant in preterm and full-term umbilical cords.

## 1. Introduction

The banking of umbilical cord tissue and its use in clinical practice has gained popularity in recent years, although research has mainly focused on mesenchymal stem cells (MSCs) [1]. Several factors cause umbilical cord tissue collection and preservation to be an attractive method for treating different diseases, i.e, the procedure is painless, noninvasive, and ethical, since the umbilical cord is considered to be a medical waste [2]. Processes such as extracellular matrix remodeling, angiogenesis, progenitor activity, and immunomodulation are targets worth exploring when discussing potential modern therapeutic approaches to various pathologies. Accordingly, the exploration of the expression of markers representing these essential events in umbilical cord tissue can further characterize MSCs and their biological properties, as well as provide an additional understanding regarding whether other umbilical cord cells, aside from MSCs, could be suitable for use in clinical practice. Also, little is known about morphogenetic changes in the umbilical cord during the maturation process, and investigation of the representative markers can improve the understanding of physiological events occurring in the umbilical cord.

Matrix metalloproteinase-2 (MMP2), also known as gelatinase A or type IV collagenase, is a zinc-dependent endopeptidase that cleaves elements of the extracellular matrix (ECM) [3]. The mRNA and the MMP-2 protein are found in various tissues, including the heart, smooth muscles, colon, kidney, urinary bladder, lungs, testis and prostate, uterine cervix, endometrium, and placenta. MMP-2 is made up of extra- and peri-cellular substrates, such as type I-VI collagen, elastin, fibronectin, proteoglycans, and cytokines, i.e., interleukin-1β [4] and monocyte chemoattractant protein-3 [5]. The intracellular activity of MMP2 has also been described [6]. MMP2 contributes to physiological, as well as pathological, events. The functions of this protein include promoting angiogenesis, facilitating cell migration and cancer cell invasion and metastasis via degradation of the basement membrane [7], and regulating endometrial remodeling during pregnancy [3]. Overall matrix metalloproteinases (MMPs) can be induced by a large number of stimuli, some of which include inflammatory cytokines, growth factors, mechanical movement, and phagocytosis. Under physiological conditions, the basal expression of matrix metalloproteinases is low, and transient increases are seen during normal matrix remodeling, including wound healing, ovulation, and uterine resorption after pregnancy [8].

In the extracellular compartment, the proteolytic functions of MMPs are affected by tissue inhibitors of metalloproteinases (TIMPs). TIMP2 is the most abundant member of the TIMP family; therefore, it plays a crucial role in maintaining the balance of the extracellular matrix remodeling [9]. TIMP2 acts as an inhibitor of multiple MMPs, namely MMP-1, -2, -3, -7, -8, -9, -11, -13, -14, -16, and -24 [10], while also taking part in the cell-surface activation of pro-MMP2 by interacting with membrane type 1-MMP. TIMP2 also inhibits angiogenesis independent of MMP inhibition [11]. However, almost nothing is known regarding changes in TIMP2 expression in umbilical cords of different ages.

CD34 is a transmembrane glycoprotein typically associated with hematopoietic stem and progenitor cells. It is also expressed by mesenchymal stromal cells, muscle satellite cells, corneal keratocytes, interstitial cells, epithelial progenitors, and vascular endothelial progenitors [12]. The possible functions of CD34 include enhancing proliferation and blocking differentiation, promoting lymphocyte adhesion, and enhancing trafficking and migration of hematopoietic cells [13]. Most endothelial cells in larger veins and arteries are CD34-negative; CD34-positive endothelial cells are found within smaller blood vessels [12]. It is also reported that CD34-positive human umbilical vein endothelial cells show an increased mRNA expression of all known tip cell markers; therefore, they might play an important role during sprouting angiogenesis [14].

Vascular endothelial growth factor (VEGF, also known as VEGF-A) is a member of the VEGF family, along with VEGF-B, VEGF-C, VEGF-D, and placental growth factor (PlGF) [15]. VEGF plays a major role in regulating vasculogenesis and angiogenesis, since its actions include inducing endothelial cell migration and invasion into the basement membrane, promoting proliferation, the formation of fenestrations, and the survival of endothelial cells [16]. The production of VEGF is stimulated by the presence of hypoxia or growth factors, i.e., transforming growth factor β (TGFβ), interleukins, or platelet-derived growth factors (PDGFs) [17], and it is expressed in nearly all vascularized adult tissues, including select endothelium [18]. Many findings broaden the possible therapeutic applications for VEGF, meaning that it could be used in pro-angiogenic therapy for various ischemic diseases, as well as for some degenerative diseases [19].

Human β-defensin 2 (HBD2) is an antimicrobial peptide and an important component of innate immunity. HBD2 provides protection against bacterial, viral, fungal, and parasitic pathogens [20]. Cationic β-defensins bind to negatively charged microbial membranes, additional pores are formed, and microbial death occurs due to the permeabilization of the membranes [21]. This peptide not only exhibits direct bactericidal action but also participates in chemotaxis and Toll-like receptor activation, resulting in the modulation of immunocompetent cell responses [22]. The expression of HBD2 is detected in several types of human epithelia such as epithelia from the skin, lungs, trachea, urogenital system [23], and gastrointestinal tract [21]. HBD2 is generally expressed at low levels in normal physiological states and is induced in response to microbial stimuli; the expression of HBD2 is stimulated by pro-inflammatory mediators or bacterial products [24]. It is suggested that HBD2 could be applicable in various inflammatory diseases as a tool for modulating the response of the immune system [20].

Consequently, the aim of the study was to determine the expression and distribution of markers MMP2, TIMP2, CD34, VEGF, and HBD2 in preterm and full-term human umbilical cord tissue.

## 2. Materials and Methods

### 2.1. Patients

The material of 17 umbilical cords, obtained during child delivery, was examined. Mothers were aged 22 to 42; gestational age (GA) ranged from 28 weeks to 40 weeks. Patients were divided into two groups: (a) very preterm and moderate preterm birth umbilical cords (gestational age 28 to 33 weeks) and (b) late preterm birth and full-term birth umbilical cords (gestational age 34 to 40 weeks). The division was based on categories of preterm birth, according to the World Health Organization, as follows: full-term infants—GA between 37 weeks and 41 week and 6 days; late preterm infants—GA between 34 weeks and 36 weeks and 6 days; moderate preterm infants—GA between 32 weeks and 33 weeks and 6 days; very preterm infants—GA < 32 weeks; extremely preterm infants—GA < 28 weeks [25]. Patients with different numbers of previous pregnancies were included in the study—umbilical cord samples were taken from five patients with a 1st pregnancy, four patients with a 2nd pregnancy, four patients with a 3rd pregnancy, and four patients with a 4th pregnancy.

All tissue samples included in the study contained intact umbilical blood vessels—two arteries and one vein—and are considered to be obtained from healthy umbilical cords. Exclusion criteria were the following: patients with vascular, insertional or cystic umbilical cord abnormalities; an abnormal twist or coil; an umbilical cord true knot; a nuchal cord; a thin cord (deficiency of Wharton’s jelly); umbilical cord prolapse; a primary umbilical cord mass; or any other umbilical cord pathology.

All experiments were conducted according to the ethical standards expressed in the 1964 Declaration of Helsinki. This study was approved by the Riga Stradins University Ethics Committee, dated 12 March 2009 (No. E-9 (2)). Written informed consent was obtained from patients in each case.

### 2.2. Microscopy

#### 2.2.1. Routine Microscopy

Umbilical cord tissue pieces were fixed in a mixture of 2% formaldehyde and 0.2% picric acid in 0.1 M phosphate buffer (pH 7.2). Tissue samples were further rinsed in Tyrode’s solution containing 10% sucrose for 12 h. Afterwards, tissue specimens were embedded in paraffin and cut into 6–7 micrometer (µm) thin tissue sections. Routine histological staining with hematoxylin and eosin was used for all samples. Each specimen was then examined using bright-field microscopy to evaluate the morphological structure of the umbilical cord.

#### 2.2.2. Immunohistochemistry

A total of 17 tissue specimens were prepared for the detection of markers CD34, MMP2, TIMP2, VEGF, and HBD2, using the biotin-streptavidin immunohistochemistry (IMH) method. The characteristics and parameters of the primary antibodies used in this study are the following: CD34 (sc-19621, mouse, working dilution 1:100, Santa Cruz Biotechnology Inc., Dallas, TX, USA); MMP2 (sc-53630, mouse, working dilution 1:100, Santa Cruz Biotechnology Inc., Dallas, TX, USA); TIMP2 (sc-21735, mouse, 1:200, Santa Cruz Biotechnology Inc., Dallas, TX, USA); VEGF (ab1316, mouse, working dilution 1:200, Abcam, Cambridge, UK); HBD2 (sc-20798, rabbit, working dilution 1:100, Santa Cruz Biotechnology Inc., Dallas, TX, USA).

The Leica DC 300F camera microscope was used for the examination of the samples under bright-field microscopy. Conventional histological photographs were captured and then analyzed using the Image-Pro Plus picture visualization program. A relative number of positive immunoreactive structures was graded semi-quantitatively. The appearance and distribution of CD34-positive cells were examined in the endothelium of the umbilical arteries and the endothelium of the umbilical vein. The appearance and distribution of MMP2, TIMP2, VEGF, and HBD2 were examined in the endothelium of the umbilical arteries, the endothelium of the umbilical vein, the blood vessel wall, the extraembryonic mesenchyme, and the amniotic epithelium. Positively stained cells were counted and then graded using a scale containing the following values: 0—no positive structures (0%); 0/+—occasional positive structures (12.5%); +—few positive structures (25%); +/++—few to moderate number of positive structures (37.5%); ++—moderate number of positive structures (50%); ++/+++—moderate to numerous number of positive structures (62.5%); +++—numerous positive structures (75%); +++/++++—numerous to an abundance of positive structures (87.5%); ++++—abundance of positive structures (100%) [26].

### 2.3. Statistics

Statistical analysis of the acquired data was performed using the SPSS Statistics 29 (IBM Company, Burbank, CA, USA) statistical program. Data were ranked as ordinal values, where 0 positive structures seen in the visual field correspond with the value of 0, occasional positive structures (0/+) correspond with the value of 0.5, few positive structures (+) correspond with the value 1.0, and so forth. The highest possible number of structures—abundance of positive structures (++++)—corresponds with the value 4.0. Non-parametric tests were used in this study. The Mann–Whitney U test was performed to detect statistically significant differences in expression of markers between the two patient groups. Spearman’s rank-order correlation coefficient was calculated for the evaluation of correlations between the different markers expressed. Spearman’s rho (r_s_) value of 0.00–0.30 was considered to be a very weak correlation; 0.30–0.50 as weak; 0.50–0.70 as moderate; 0.70–0.90 as strong; and 0.90–1.00 as very strong. In the case of a strong positive correlation, the rs value is closer to +1; in the case of a strong negative correlation, the r_s_ value is closer to −1. In all cases, values of *p* < 0.05 were considered statistically significant.

## 3. Results

### 3.1. Routine Histology

**Figure 1:**
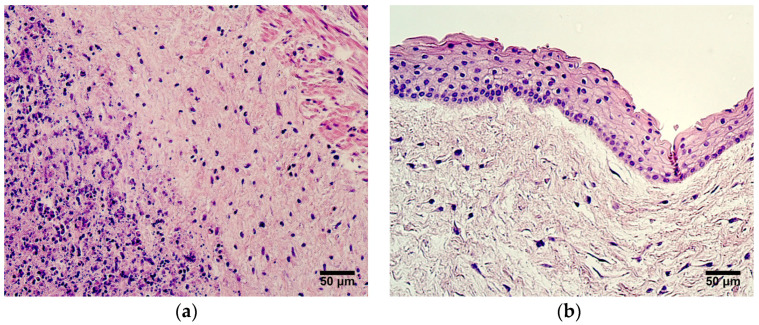
(**a**) Inflammatory infiltrate seen in Wharton’s jelly and wall of an umbilical vein, gestational age—30 weeks; hematoxylin and eosin, X200. (**b**) Multiple layers of amniotic epithelium cells, gestational age—36 weeks; hematoxylin and eosin, X200.

The inflammatory infiltrate in Wharton’s jelly and in the walls of the blood vessels was observed in two patient samples from the very preterm and moderate preterm birth umbilical cord group (Figure 1a), as well as in two patient samples from the late preterm birth and full-term birth umbilical cord group. In four samples, zones with multiple layers of amniotic epithelium cells could be visualized (Figure 1b). No other changes from the previously described well-known typical structures of umbilical cords were seen.

### 3.2. Immunohistochemistry

**Figure 2:**
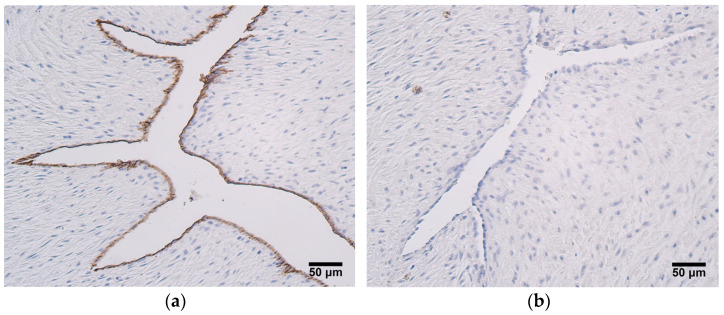
(**a**) Numerous CD34-positive cells in endothelium of an umbilical artery, gestational age—28 weeks; CD34 IMH, X200. (**b**) No CD34-positive cells are observed in endothelium and blood vessel wall of an umbilical artery, gestational age—40 weeks; CD34 IMH, X200.

The highest relative number of CD34-positive cells, which are numerous positive structures, was seen in the endothelium of the umbilical arteries in very preterm and moderate preterm birth umbilical cords (Figure 2a). The relative number of CD34-positive cells in the endothelium of the umbilical vein in very preterm and moderate preterm birth umbilical cords varied, where half of the samples contained occasional or more positive structures, while other half contained no positive structures. Most late preterm birth and full-term birth umbilical cords did not contain any CD34-positive endothelial cells in either the arteries or veins (Figure 2b, Table 1). The blood vessel wall, extraembryonic mesenchyme, and amniotic epithelium were CD34-negative in all cases.

**Figure 3:**
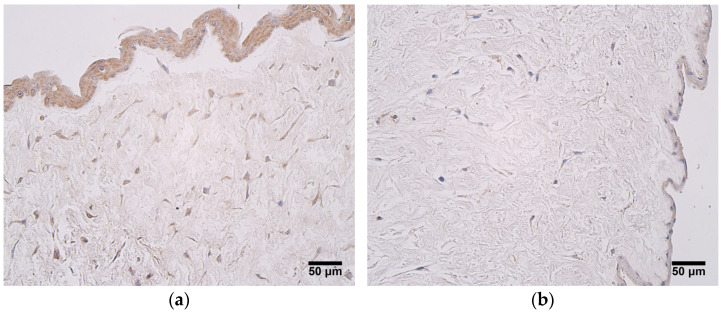

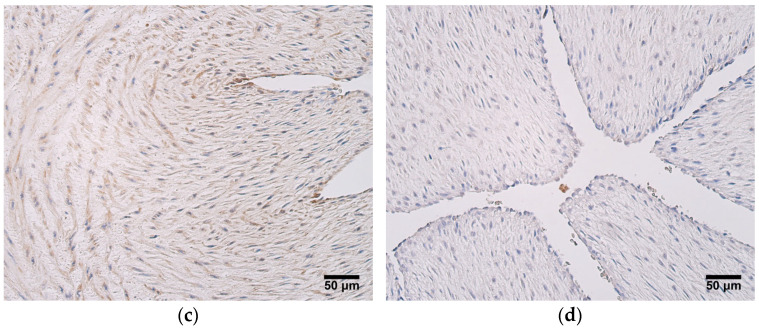
(**a**) Numerous MMP2-positive cells in extraembryonic mesenchyme and amniotic epithelium, gestational age—33 weeks; MMP2 IMH, X200. (**b**) Moderate number of MMP2-positive cells in extraembryonic mesenchyme and amniotic epithelium, gestational age—40 weeks; MMP2 IMH, X200. (**c**) Few MMP2-positive cells in endothelium of an umbilical artery and few to moderate number of MMP2-positive cells in the wall of an umbilical artery, gestational age—32 weeks; MMP2 IMH, X200. (**d**) No MMP2-positive cells are seen in endothelium and wall of an umbilical artery, gestational age—40 weeks; MMP2 IMH, X200.

The strongest expression of MMP2 was observed in extraembryonic mesenchyme and in the amniotic epithelium of both very preterm and moderate preterm birth umbilical cords (in most cases, numerous positive cells were found) (Figure 3a), as well as in late preterm and full-term birth umbilical cords (in most cases, a moderate number of positive cells was found) (Figure 3b). Compared with late preterm and full-term birth umbilical cords, the endothelium of the umbilical vein and the blood vessel wall of very preterm and moderate preterm birth umbilical cords contained more MMP2-positive cells, ranging from zero to few positive structures in the endothelium of the umbilical vein. The number of positive cells in the blood vessel wall of the same samples ranged from zero, to few, to moderate (Figure 3c). The endothelium of the umbilical vessels and the blood vessel wall in late preterm and full-term birth umbilical cords mainly contained zero MMP2-positive cells (Figure 3d), (Table 2).

**Figure 4:**
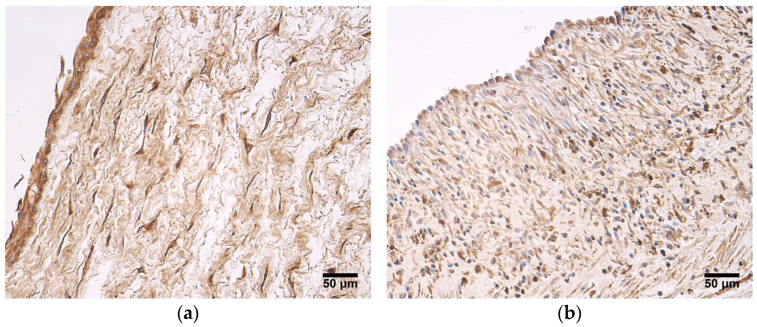
(**a**) Numerous to an abundance of TIMP2-positive cells are seen in extraembryonic mesenchyme and in amniotic epithelium, gestational age—30 weeks; TIMP2 IMH, X200. (**b**) Numerous TIMP2-positive cells are seen in endothelium and wall of an umbilical vein, gestational age—28 weeks; TIMP2 IMH, X200.

The expression of TIMP2 was noteworthy in both patient groups and in all tissues examine; however, the extraembryonic mesenchyme of very preterm and moderate preterm birth umbilical cords showed the highest relative number of TIMP2-positive cells, with the modal value being numerous to an abundance of positive structures seen (Figure 4a). The lowest expression of TIMP2 was found in the endothelium of the umbilical arteries of late preterm birth and full-term birth umbilical cords, where a moderate number of positive structures was most frequently observed. The endothelium of the umbilical vein, however, showed mostly numerous positive cells in both patient groups (Figure 4b), (Table 3).

**Figure 5:**
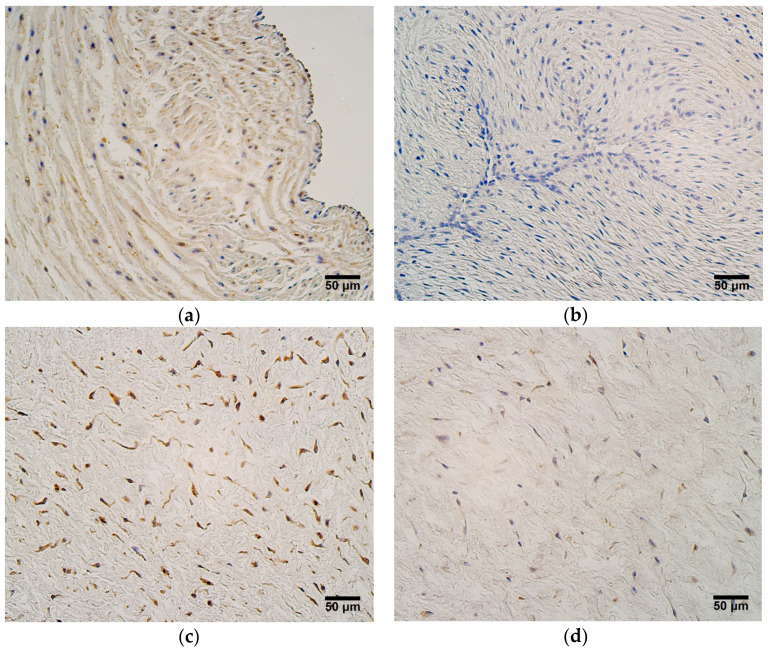
(**a**) Moderate number of VEGF-positive cells is seen in endothelium and wall of an umbilical vein, gestational age—33 weeks; VEGF IMH, X200. (**b**) No VEGF-positive cells are seen in endothelium and wall of an umbilical artery, gestational age—40 weeks; VEGF IMH, X200. (**c**) Numerous VEGF-positive cells can be found in extraembryonic mesenchyme, gestational age—28 weeks; VEGF IMH, X200. (**d**) Moderate number of VEGF-positive cells is seen in extraembryonic mesenchyme, gestational age—40 weeks; VEGF IMH, X200.

The expression of VEGF was similar in both patient groups, and equal modal values of VEGF expression were seen in the endothelium of the umbilical arteries, the endothelium of the umbilical vein, the extraembryonic mesenchyme, and the amniotic epithelium among the two groups. The expression of VEGF was the strongest in the extraembryonic mesenchyme, and numerous positive cells were found in most samples (Figure 5c), although three patients from the late preterm birth and full-term birth umbilical cord group showed a moderate expression of VEGF in the extraembryonic mesenchyme (Figure 5d). The lowest number of VEGF-positive cells was found in the endothelium of the umbilical arteries, with zero positive structures seen in visual field as the prevailing value (Figure 5b). The most evident differences in VEGF expression between the two patient groups were noticed in the blood vessel wall; very preterm and moderate preterm birth umbilical cords contained mostly a moderate number of positive cells (Figure 5a) in contrast to the late preterm birth and full-term birth umbilical cords, where a moderate number of VEGF-positive cells could be visualized in only one case (Table 4).

**Figure 6:**
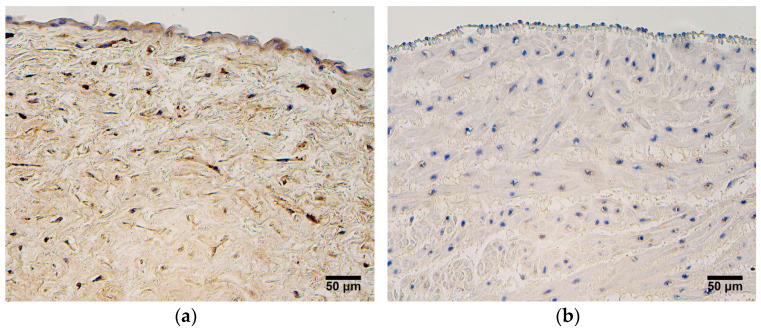
(**a**) Moderate number of HBD2-positive cells is found in amniotic epithelium, and numerous HBD2-positive cells are found in extraembryonic mesenchyme, gestational age—30 weeks; HBD2 IMH, X200. (**b**) No HBD2-positive cells are seen in endothelium and blood vessel wall of an umbilical vein, gestational age—38 weeks; HBD2 IMH, X200.

In both patient groups, the expression of HBD2 was the highest in the extraembryonic mesenchyme and amniotic epithelium, where a mostly moderate number of HBD2-positive cells was seen (Figure 6a). Three samples of very preterm and moderate preterm birth umbilical cords showed numerous positive cells in the extraembryonic mesenchyme (Figure 6a). The endothelium of the umbilical arteries and veins, as well as the blood vessel wall, did not show a strong expression of HBD2 in very preterm and moderate preterm birth umbilical cords, nor was there a strong expression in the late preterm birth and full-term birth umbilical cords; in most samples, no HBD2-positive cells were seen (Figure 6b), (Table 5).

### 3.3. Statistical Data

A Mann–Whitney U test showed statistically significant differences in the expression of the following markers between very preterm and moderate preterm birth umbilical cords and late preterm and full-term birth umbilical cords: CD34 in the endothelium of umbilical arteries (*U* = 11.0, *p* = 0.028); MMP2 in the amniotic epithelium (*U* = 8.5, *p* = 0.010); TIMP2 in the blood vessel wall (*U* = 14.5, *p* = 0.036); TIMP2 in the extraembryonic mesenchyme (*U* = 13.0, *p* = 0.027); TIMP2 in the amniotic epithelium (*U* = 11.5, *p* = 0.028) (Table 6).

Multiple strong and very strong correlations between the studied markers were found in very preterm and moderate preterm birth umbilical cords. Interestingly, the strongest positive correlation was observed between the expression of CD34 in the endothelium of the umbilical vein and the expression of HBD2 in the amniotic epithelium (r_s_ = 0.993, *p* < 0.001). Another very strong positive correlation was detected between MMP2-positive cells in the endothelium of the umbilical arteries and TIMP2-positive cells in the amniotic epithelium (r_s_ = 0.932, *p* < 0.001). In total, six strong and one very strong correlation was seen between different umbilical cord tissue compartments expressing VEGF. Additionally, eleven strong correlations were noted between the expression of CD34 and MMP2; CD34 and TIMP2; MMP2 and TIMP2; MMP2 and HBD2; MMP2 and VEGF; and TIMP2 expression in different umbilical cord compartments (Table 7).

Multiple strong and very strong correlations between the studied markers were also noted in late preterm and full-term birth umbilical cords. The strongest correlation was found between MMP2-positive and TIMP2-positive cells in the extraembryonic mesenchyme (*r_s_* = 0.937, *p* < 0.001). Interestingly, four strong positive correlations were detected between HBD2-positive cells in the amniotic epithelium and VEGF-positive cells in various tissue compartments. Four strong positive correlations could be observed between TIMP2-expressing and VEGF-expressing cells in various tissue compartments. In total, seven strong and one very strong correlation was seen between different umbilical cord tissue compartments expressing VEGF. Additionally, eight strong correlations were noted between the expression of MMP2 and VEGF; MMP2 and HBD2; TIMP2 and HBD2; and TIMP2 expression in different umbilical cord compartments (Table 8).

## 4. Discussion

In this study, we found that the expression of MMP2 was more pronounced in very preterm and moderate preterm birth umbilical cords than in late preterm birth and full-term birth umbilical cords. MMP2 was mainly produced in the amniotic epithelium and the extraembryonic mesenchyme. Statistically significant differences in MMP2 expression between the two groups were seen in the amniotic epithelium. These findings suggest that the MMP2 activity decreases with an increase in the umbilical cord’s age. MMPs play a major role in the degradation of ECM, subsequently affecting processes such as cell migration, adhesion, and differentiation. ECM remodeling is prominent during organogenesis [27]. MMP-2 also mediates ribosomal RNA transcription by cleaving nucleolar histones and it plays a role in cell proliferation [28]. Research shows that the number of MMP2s found in umbilical cord vessels is several times higher than that of other MMPs, and pre-eclampsia causes a decrease in MMP2 levels [29]. The reduction of MMP2 expression in late preterm birth and full-term birth umbilical cords could be explained with the proposition that ECM remodeling is not as essential at this stage as it is in earlier weeks of umbilical cord development. The findings of this study are in accordance withthose of other researchers. A different study also reported the expression of MMP2 in umbilical cord epithelial cells and in Wharton’s jelly fibroblasts [30]. Interestingly, Mauro et al. [30] revealed that MMP2 expression was found in cultured human umbilical vein endothelial cells, while it was not detected in the whole umbilical cord. The authors of said study suggest that blood vessel endothelium is a fragile structure, and that it could easily be damaged during the fixation and embedding procedures used when performing the immunohistochemistry assays. This possibility cannot be excluded in our study, since approximately half of the samples examined did not show the expression of MMP2 in the endothelium of the umbilical vein. MMP2 can take part in pathological processes as well. It can be considered a prognostic factor for several tumors—overexpression of MMP2 is associated with poor prognosis in oral cancers, retinoblastoma, bladder cancer, epithelial cancer, and breast cancer [31]. It is also proposed that MMP2 may be involved in ECM degradation and placental barrier dysfunction, leading to pathogen transmission [32]; therefore, while it is an important part of maintaining the physiological remodeling processes in the umbilical cord, its overexpression should be viewed with caution. 

One factor that opposes the actions of MMP2 is TIMP2, and its presence was noteworthy in all umbilical cord tissue compartments in both patient groups. Statistically significant differences in TIMP2 expression between very preterm and moderate preterm birth umbilical cords and late preterm birth and full-term birth umbilical cords were seen in the blood vessel wall, the extraembryonic mesenchyme, and the amniotic epithelium; greater TIMP2 expression levels were observed in very preterm and moderate preterm birth umbilical cords. A very strong correlation was also found between TIMP2 and MMP2 expression in both patient groups, revealing that ECM remodeling in the umbilical cord is strongly regulated. When compared with the number of MMP2-positive cells, in the umbilical cord, TIMP2 shows overall greater expression. TIMP2 is known to form complexes with other MMPs aside from just MMP2 [33]; at the same time, other MMPs, such as MMP9, can also be found in umbilical cord tissue [30]. For that reason, it is possible that a considerable amount of TIMP2 is necessary to maintain tissue homeostasis in the umbilical cord. It is important to note that TIMP2 can also have stimulatory effects by interacting with pro-MMP2 and forming a trimolecular complex with MMP14, further participating in activation of MMP2. It is suggested that after this cascade, TIMP2 can be either degraded or recycled as an intact TIMP2 molecule [9]. In quiescent adult tissues, sources of TIMP2 include stromal fibroblasts, perivascular smooth muscle, and endothelial cells. In such an environment, TIMP2 inhibits endothelial cell responses to minor fluctuations in angiogenic growth factors by binding to available α3β1-integrin receptors on the endothelial cells. In a situation where angiogenic factor concentrations rise due to hypoxia or other pathological stimuli, endothelial cells increase the production of MMPs and decrease the synthesis of TIMP2, while simultaneously, excess TIMP2 binds to proteases, leading to a reduction in local TIMP2 concentration [11]. We speculate that this model of TIMP2 action could also be applicable to the human umbilical cord, where inhibitory activity dominates with regard to ECM turnover, including the appearance of angiogenesis.

In this study, the extraembryonic mesenchyme contained more VEGF-positive cells than did other tissue compartments in both the very preterm and moderate preterm birth umbilical cords and the late preterm birth and full-term birth umbilical cords. The number of VEGF-positive cells in the endothelium of the umbilical arteries and veins and the blood vessel wall varied, suggesting the presence of some individual factor affecting VEGF expression in these structures. A potent stimulus for VEGF expression is hypoxia; however, several other factors can participate in VEGF induction as well. Fibroblast growth factor, transforming growth factors (TGF-α and TGF-β), keratinocyte growth factor, insulin-like growth factor 1 (IGF-1), platelet-derived growth factor, as well as the inflammatory cytokines interleukin IL-1α and IL-6, can all up-regulate VEGF expression [34]. No statistically significant differences in VEGF expression were detected between the two patient groups, suggesting that the VEGF activity in the umbilical cord is constant throughout the pregnancy. There have been reports that umbilical VEGF levels are higher in preterm births [35]; however, the distinction of whether these differences account for individual factors or the age of the umbilical cord still needs to be confirmed. While most commonly known as an angiogenesis promoting factor, VEGF mediates other crucial functions as well, such as the reduction of apoptosis in endothelial cells [36]. Having this essential purpose—the maintenance of endothelial cell survival—might explain why all samples examined in this study showed VEGF-positive cells in the extraembryonic mesenchyme, independent of the umbilical cord’s age. The expression pattern observed in this study is in agreement with those noted by previous researchers, where VEGF was also detected in the endothelial, stromal, and amniotic cells of the umbilical cord [37]. In addition to its physiological properties, VEGF is thought to contribute to the pathogenesis of preeclampsia by disturbing the umbilical cord vascular endothelium, where its expression is significantly elevated [38]. While VEGF is a common and essential component of umbilical cord tissue, its signalling must not be disregulated. 

In this study, CD34-positivity was found only in the endothelium of the umbilical arteries and veins. The most prominent expression of CD34 was seen in the endothleium of the umbilical arteries in very preterm and moderate preterm birth umbilical cords, and statistically significant differences in CD34 expression between the two patient groups were also detected. It is proposed that CD34 can be considered a marker of subpopulations of progenitor cells from larger non-hematopoietic cell populations [12], and it could be possible that this progenitor phenotype is lost with an increase in the umbilical cord’s age. Functional endothelial precursor cells (EPC) are characterized by the presence of the following markers—CD34, CD133, and vascular endothelial growth factor receptor-2 (VEGFR-2). More mature and differentiated EPCs are found in peripheral circulation, and they show a decreased expression of CD133. Finally, mature endothelial cells located in large vessels are CD34-negative [39]. In only a few samples of this study did the umbilical cord vein endothelial cells stain positive for CD34. It has been reported that mature human umbilical vein endothelial cells are CD133-negative; thus, a loss of CD133 and subsequently, of CD34, can be observed during the transformation from an EPC to a mature endothelial cell [39]. Previous research shows that CD34-positive human umbilical vein endothelial cells possess the properties of tip cells, which are essential components of sprouting angiogenesis. In addition, the number of CD34-positive cells increased upon stimulation with VEGF in vitro [14]. We hypothesize that human umbilical artery endothelial cells could also carry the characteristics of tip cells, since in this research, CD34 expression was more noteable in the umbilical arteries.

The expression of HBD2 was consistently seen in the extraembryonic mesenchyme and the amniotic epithelium of very preterm and moderate preterm birth umbilical cords, as well as in late preterm birth and full-term birth umbilical cords. No significant differences were found in HBD2 expression among the two patient groups. These findings suggest that the presence of antimicrobial molecules in the umbilical cord are consistent throughout pregnancy, and that these molecules are mainly produced outside of umbilical vessels. It is known that HBD2 plays a crucial role in mesenchymal stem cell-mediated microbicidal effects [40]. In addition, HBD2 might play a role in protection against the uncontrolled activation of the complement system by inhibiting the classical pathway [41]. In 2013, Olbrich et al. [42] reported that HBD2 serum levels were lower in preterm neonates than in term infants, and that low HBD2 levels might be an independent risk factor for late-onset sepsis. It can be speculated that this HBD2 deficiency in preterm neonates arises from a region different than the umbilical cord, since the expression of HBD2 in umbilical cord tissues was similar in differently aged umbilical cords. Interestingly, a very strong correlation was found between the expression of CD34 in the endothelium of the umbilical vein and the expression of HBD2 in the amniotic epithelium. CD34-positive endothelial progenitor cells are thought to participate in NO-dependent vasodilatation and hyperpermeability [43]. Research suggests that HBD2 opens Ca^2+^-activated potassium channels and induces vasodilation in monkeys [44]; therefore, the two markers—CD34 and HBD2—could be cooperating in modulating vascular function.

All things considered, in umbilical cords of different ages, VEGF and HBD2 were expressed in a constant manner, while MMP2, TIMP2, and CD34 were seen more frequently in very preterm and moderate preterm birth umbilical cords. These findings reveal that VEGF and HBD2 are stable markers when compared among very preterm and moderate preterm birth umbilical cords and late preterm birth and full-term birth umbilical cords. MMP2, TIMP2, and CD34, however, might play a role that is especially important during earlier periods of umbilical cord maturation.

This study exhibits a few limitations, such as relatively small number of patients and a lack of information about clinical outcomes. A larger number of study samples would contribute to more representative results, as well as allow for dividing the patients into smaller groups, perhaps observing a treshold of the umbilical cord’s age for change in certain marker expression levels. This study could be expanded by evaluating maternal factors that might affect specific marker expression, as well as by following the clinical outcomes with regard to specific marker levels in the umbilical cord. Tissue factor levels could also be further characterized using enzyme-linked immunosorbent assay (ELISA), leading to fully quantitative results. The evaluation of gene expression can provide further insight into the pysiological function of the umbilical cord; thus, mRNA levels could be assessed. Additional extension of this research could be achieved by exploring the possible association of tissue factor expression in the umbilical cord with the placental health status and the molecular and immunohistochemical qualities of placenta, as well as by detecting the expression different genes and gene proteins that might affect the development of the umbilical cord and placental vessels, i.e., placental growth factor and angiopoietins [45].

## 5. Conclusions

Extracellular matrix remodeling in very preterm and moderate preterm birth umbilical cords and late preterm birth and full-term birth umbilical cords is strongly regulated, and tissue factors MMP2 and TIMP2 take part in this process by showing the decrease in MMP2 and the consistent increase in TIMP2 positive cells.

The expression of VEGF is not changed by the umbilical cord’s age; however, individual patient factors can affect the production of VEGF, suggesting the constant suppression of ischemia throughout the placenta development.

Numerous CD34-positive cells in the endothelium of the umbilical arteries suggest a significant role of progenitor cells in very preterm and moderate preterm birth umbilical cords.

Antimicrobial activity provided by HBD2 is essential and constant in very preterm and moderate preterm birth umbilical cords, as well as in late preterm birth and full-term birth umbilical cords.

The extraembryonic mesenchyme cells are the most active cell producers, expressing MMP2, TIMP2, VEGF, and HBD2 at notable levels in very preterm and moderate preterm birth umbilical cords, as well as in late preterm birth and full-term birth umbilical cords.

## Figures and Tables

**Table 1:** Expression of CD34 in endothelium of human umbilical vein and artery, depending on gestational age.

Specimen Number	Endothelium of Umbilical Arteries	Endothelium of Umbilical Vein
Very preterm and moderate preterm birth umbilical cords		
1 *	0	0
2 *	+++ (3)	0
3	+++ (3)	++ (2)
4	+++ (3)	0/+ (0.5)
5	++/+++ (2.5)	0/+ (0.5)
6	+++ (3)	+ (1)
7	0/+ (0.5)	0
8	0/+ (0.5)	0
Modal value	+++ (3)	0
Late preterm birth and full-term birth umbilical cords		
1	0	0
2	0	0
3	0	0
4	0	0/+ (0.5)
5	+ (1)	0
6	+/++ (1.5)	0
7	0/+ (0.5)	0
8 *	++/+++ (2.5)	0
9 *	0	0
Modal value	0	0

Abbreviations: 0—no positive structures (0%); 0/+—occasional positive structures (12.5%); +—few positive structures (25%); +/++—few to moderate number of positive structures (37.5%); ++—moderate number of positive structures (50%); ++/+++—moderate to numerous number of positive structures (62.5%); +++—numerous positive structures (75%); *—samples with inflammatory infiltrate seen under light microscopy.

**Table 2:** Expression of MMP2 in human umbilical cord tissue, depending on gestational age.

Specimen Number	Endothelium of Umbilical Arteries	Endothelium of Umbilical Vein	Blood Vessel Wall	Extraembryonic Mesenchyme	Amniotic Epithelium
Very preterm and moderate preterm birth umbilical cords					
1 *	0	0	0	+++ (3)	+++ (3)
2 *	+ (1)	0/+ (0.5)	+/++ (1.5)	++/+++ (2.5)	+++ (3)
3	+ (1)	+ (1)	0/+ (0.5)	++/+++ (2.5)	+++ (3)
4	0	0/+ (0.5)	0/+ (0.5)	+++ (3)	++/+++ (2.5)
5	0	+ (1)	0	+++ (3)	+++ (3)
6	+ (1)	+ (1)	+/++ (1.5)	+++ (3)	+++ (3)
7	0/+ (0.5)	0	0	+++ (3)	++/+++ (2.5)
8	0	0	+/++ (1.5)	+++ (3)	++ (2)
Modal value	0	0; + (1)	0; +/++ (1.5)	+++ (3)	+++ (3)
Late preterm and full-term birth umbilical cords					
1	0	+ (1)	0	++/+++ (2.5)	++ (2)
2	0	0/+ (0.5)	0	+++ (3)	++ (2)
3	+ (1)	+ (1)	0	++ (2)	++ (2)
4	0	0	0	++ (2)	-
5	0/+ (0.5)	+ (1)	0/+ (0.5)	+++ (3)	++ (2)
6	0	0	0	+++ (3)	+++ (3)
7	0	0	0	++ (2)	++ (2)
8 *	0	0	0/+ (0.5)	++ (2)	++ (2)
9 *	0	0	0	+/++ (1.5)	+/++ (1.5)
Modal value	0	0	0	++ (2)	++ (2)

Abbreviations: 0—no positive structures (0%); 0/+—occasional positive structures (12.5%); +—few positive structures (25%); +/++—few to moderate number of positive structures (37.5%); ++—moderate number of positive structures (50%); ++/+++—moderate to numerous number of positive structures (62.5%); +++—numerous positive structures (75%); MMP2—matrix metalloproteinase 2; *—samples with inflammatory infiltrate observed under light microscopy.

**Table 3:** Expression of TIMP2 in human umbilical cord tissue, depending on gestational age.

Specimen Number	Endothelium of Umbilical Arteries	Endothelium of Umbilical Vein	Blood Vessel Wall	Extraembryonic Mesenchyme	Amniotic Epithelium
Very preterm and moderate preterm birth umbilical cords					
1 *	++/+++ (2.5)	+++ (3)	++/+++ (2.5)	+++/++++ (3.5)	++/+++ (2.5)
2 *	+++ (3)	+++ (3)	+++ (3)	+++/++++ (3.5)	+++/++++ (3.5)
3	++/+++ (2.5)	+++ (3)	+++ (3)	+++/++++ (3.5)	+++/++++ (3.5)
4	+/++ (1.5)	+++ (3)	+++ (3)	++++ (4)	+++ (3)
5	0	++ (2)	++ (2)	+++/++++ (3.5)	++/+++ (2.5)
6	+++ (3)	+++ (3)	+++ (3)	++++ (4)	+++/++++ (3.5)
7	++ (2)	+++ (3)	+++ (3)	+++/++++ (3.5)	+++ (3)
8	++ (2)	++ (2)	++/+++ (2.5)	+++/++++ (3.5)	++/+++ (2.5)
Modal value	++ (2); ++/+++ (2.5); +++ (3)	+++ (3)	+++ (3)	+++/++++ (3.5)	++/+++ (2.5); +++/++++ (3.5)
Late preterm birth and full-term birth umbilical cords					
1	0/+ (0.5)	++ (2)	+ (1)	+++ (3)	++ (2)
2	++ (2)	+++ (3)	++/+++ (2.5)	+++/++++ (3.5)	++/+++ (2.5)
3	++ (2)	++ (2)	++/+++ (2.5)	+++ (3)	+++ (3)
4	0	+ (1)	++ (2)	+++ (3)	-
5	++ (2)	+++ (3)	++/+++ (2.5)	++++ (4)	++/+++ (2.5)
6	0/+ (0.5)	0/+ (0.5)	++ (2)	+++/++++ (3.5)	++/+++ (2.5)
7	++ (2)	+++ (3)	++/+++ (2.5)	+++ (3)	++/+++ (2.5)
8 *	++/+++ (2.5)	++/+++ (2.5)	+++ (3)	+++ (3)	++/+++ (2.5)
9 *	++ (2)	0	+ (1)	++ (2)	++ (2)
Modal value	++ (2)	+++ (3)	++/+++ (2.5)	+++ (3)	++/+++ (2.5)

Abbreviations: 0—no positive structures (0%); 0/+—occasional positive structures (12.5%); +—few positive structures (25%); +/++—few to moderate number of positive structures (37.5%); ++—moderate number of positive structures (50%); ++/+++—moderate to numerous number of positive structures (62.5%); +++—numerous positive structures (75%); +++/++++—numerous to abundance of positive structures (87.5%); ++++—abundance of positive structures (100%); TIMP2—tissue inhibitor of metalloproteinases 2; *—samples with inflammatory infiltrate observed under light microscopy.

**Table 4:** Expression of VEGF in human umbilical cord tissue, depending on gestational age.

Specimen Number	Endothelium of Umbilical Arteries	Endothelium of Umbilical Vein	Blood Vessel Wall	Extraembryonic Mesenchyme	Amniotic Epithelium
Very preterm and moderate preterm birth umbilical cords					
1 *	0	0	0/+ (0.5)	++ (2)	++ (2)
2 *	++ (2)	++ (2)	++ (2)	+++ (3)	++/+++ (2.5)
3	0	+ (1)	+/++ (1.5)	+++ (3)	++ (2)
4	+ (1)	+/++ (1.5)	++ (2)	+++/++++ (3.5)	++ (2)
5	0	+ (1)	0	++ (2)	++ (2)
6	++/+++ (2.5)	++ (2)	++/+++ (2.5)	+++/++++ (3.5)	+++ (3)
7	+ (1)	++ (2)	++ (2)	+++ (3)	++/+++ (2.5)
8	++ (2)	++ (2)	++ (2)	+++ (3)	++/+++ (2.5)
Modal value	0	++ (2)	++ (2)	+++ (3)	++ (2)
Late preterm birth and full-term birth umbilical cords					
1	0	0/+ (0.5)	0	++ (2)	0
2	++ (2)	++ (2)	++ (2)	+++ (3)	+++/++++ (3.5)
3	0	0	0/+ (0.5)	++ (2)	++ (2)
4	0	0	0	+ (1)	0
5	+ (1)	++ (2)	0/+ (0.5)	+++ (3)	++ (2)
6	+ (1)	+/++ (1.5)	+/++ (1.5)	+++/++++ (3.5)	+++/++++ (3.5)
7	++/+++ (2.5)	++ (2)	+ (1)	+++ (3)	++ (2)
8 *	+ (1)	+ (1)	+ (1)	++ (2)	++ (2)
9 *	0	0	0	+ (1)	++ (2)
Modal value	0	0; ++ (2)	0	++ (2); +++ (3)	++ (2)

Abbreviations: 0—no positive structures (0%); 0/+—occasional positive structures (12.5%); +—few positive structures (25%); +/++—few to moderate number of positive structures (37.5%); ++—moderate number of positive structures (50%); ++/+++—moderate to numerous number of positive structures (62.5%); +++—numerous positive structures (75%); +++/++++—numerous to abundance of positive structures (87.5); VEGF—vascular endothelial growth factor; *—samples with inflammatory infiltrate observed under light microscopy.

**Table 5:** Expression of HBD2 in human umbilical cord tissue, depending on gestational age.

Specimen Number	Endothelium of Umbilical Arteries	Endothelium of Umbilical Vein	Blood Vessel Wall	Extraembryonic Mesenchyme	Amniotic Epithelium
Very preterm and moderate preterm birth umbilical cords					
1 *	0	0	0	++ (2)	++ (2)
2 *	0/+ (0.5)	0/+ (0.5)	++ (2)	++ (2)	++ (2)
3	0	0	0	+++ (3)	+++ (3)
4	+ (1)	0	0	++ (2)	++/+++ (2.5)
5	0	0/+ (0.5)	0	+++ (3)	++/+++ (2.5)
6	0	0	0	++/+++ (2.5)	+++ (3)
7	0	+/++ (1.5)	0	+++ (3)	++ (2)
8	0	0	0	+/++ (1.5)	++ (2)
Modal value	0	0	0	++ (2); +++ (3)	++ (2)
Late preterm birth and full-term birth umbilical cords					
1	0	0/+ (0.5)	0	++ (2)	+ (1)
2	0	0	0	++ (2)	++ (2)
3	0	0	0	++ (2)	+/++ (1.5)
4	0	0	0	++ (2)	-
5	0	0	0	++ (2)	++ (2)
6	0	0	0	+++ (3)	+++ (3)
7	0	0	0	++ (2)	++ (2)
8 *	0/+ (0.5)	0/+ (0.5)	0	++ (2)	++ (2)
9 *	0	0	0	++ (2)	0
Modal value	0	0	0	++ (2)	++ (2)

Abbreviations: 0—no positive structures (0%); 0/+—occasional positive structures (12.5%); +—few positive structures (25%); +/++—few to moderate number of positive structures (37.5%); ++—moderate number of positive structures (50%); ++/+++—moderate to numerous number of positive structures (62.5%); +++—numerous positive structures (75%); HBD2—human beta defensin 2; *—samples with inflammatory infiltrate observed under light microscopy.

**Table 6:** Significant differences in marker expression between very preterm and moderate preterm birth umbilical cords and late preterm and full-term birth umbilical cords.

Marker and Tissue Compartment	*U* Value	*p* Value
CD34 (endothelium of umbilical arteries)	11.0	0.028
MMP2 (amniotic epithelium)	8.5	0.010
TIMP2 (blood vessel wall)	14.5	0.036
TIMP2 (extraembryonic mesenchyme)	13.0	0.027
TIMP2 (amniotic epithelium)	11.5	0.028

Abbreviations: *U*—Mann–Whitney U test; *p* value—*p* values < 0.05 are considered statistically significant; MMP2—matrix metalloproteinase-2; TIMP2—tissue inhibitor of metalloproteinases-2.

**Table 7:** Summary of Spearman’s rank-order correlation analysis to determine the strong (r_s_ = 0.7–0.9) and very strong (r_s_ = 0.9–1.0) relationship between the number of marker-containing cells in different tissue compartments in very preterm and moderate preterm birth umbilical cords.

Compared Markers and Tissue Compartments		*n*	r_s_	*p* Value
Very strong correlation (r_s_ = 0.9–1.0)				
CD34 (endothelium of umbilical vein)	HBD2 (amniotic epithelium)	8	0.993	<0.001
MMP2 (endothelium of umbilical arteries)	TIMP2 (amniotic epithelium)	8	0.932	<0.001
VEGF (endothelium of umbilical arteries)	VEGF (blood vessel wall)	8	0.921	0.001
Strong correlation (r_s_ = 0.7–0.9)				
CD34 (endothelium of umbilical arteries)	MMP2 (endothelium of umbilical vein)	8	0.743	0.035
CD34 (endothelium of umbilical arteries)	TIMP2 (amniotic epithelium)	8	0.811	0.015
CD34 (endothelium of umbilical vein)	MMP2 (endothelium of umbilical vein)	8	0.878	0.004
MMP2 (endothelium of umbilical arteries)	TIMP2 (endothelium of umbilical arteries)	8	0.770	0.025
MMP2 (endothelium of umbilical arteries)	TIMP2 (blood vessel wall)	8	0.723	0.043
MMP2 (endothelium of umbilical vein)	HBD2 (amniotic epithelium)	8	0.885	0.004
MMP2 (blood vessel wall)	VEGF (endothelium of umbilical arteries)	8	0.817	0.013
TIMP2 (endothelium of umbilical vein)	TIMP2 (blood vessel wall)	8	0.800	0.017
TIMP2 (blood vessel wall)	TIMP2 (amniotic epithelium)	8	0.873	0.005
TIMP2 (blood vessel wall)	VEGF (extraembryonic mesenchyme)	8	0.757	0.030
TIMP2 (extraembryonic mesenchyme)	VEGF (extraembryonic mesenchyme)	8	0.816	0.013
VEGF (endothelium of umbilical arteries)	VEGF (endothelium of umbilical vein)	8	0.895	0.003
VEGF (endothelium of umbilical arteries)	VEGF (amniotic epithelium)	8	0.900	0.002
VEGF (endothelium of umbilical vein)	VEGF (blood vessel wall)	8	0.850	0.007
VEGF (endothelium of umbilical vein)	VEGF (amniotic epithelium)	8	0.895	0.003
VEGF (blood vessel wall)	VEGF (extraembryonic mesenchyme)	8	0.863	0.006
VEGF (blood vessel wall)	VEGF (amniotic epithelium)	8	0.827	0.011

Abbreviations: n—pairs analysed in Spearman’s rank-order correlation analysis; r_s_—correlation coefficient (Spearman’s rho); *p* value—*p* values < 0.05 are considered statistically significant; MMP2—matrix metalloproteinase-2; TIMP2—tissue inhibitor of metalloproteinases-2; VEGF—vascular endothelial growth factor; HBD2—human beta defensin-2.

**Table 8:** Summary of Spearman’s rank-order correlation analysis to determine the strong (r_s_ = 0.7–0.9) and very strong (r_s_ = 0.9–1.0) relationship between the number of marker-containing cells in different tissue compartments in late preterm and full-term birth umbilical cords.

Compared Markers and Tissue Compartments		*n*	r_s_	*p* Value
Very strong correlation (r_s_ = 0.9–1.0)				
MMP2 (extraembryonic mesenchyme)	TIMP2 (extraembryonic mesenchyme)	9	0.937	<0.001
VEGF (endothelium of umbilical arteries)	VEGF (endothelium of umbilical vein)	9	0.918	<0.001
Strong correlation (r_s_ = 0.7–0.9)				
MMP2 (extraembryonic mesenchyme)	VEGF (extraembryonic mesenchyme)	9	0.779	0.013
MMP2 (amniotic epithelium)	HBD2 (extraembryonic mesenchyme)	8	0.756	0.030
MMP2 (amniotic epithelium)	HBD2 (amniotic epithelium)	8	0.814	0.014
MMP2 (amniotic epithelium)	VEGF (extraembryonic mesenchyme)	9	0.803	0.016
TIMP2 (endothelium of umbilical arteries)	TIMP2 (blood vessel wall)	9	0.716	0.030
TIMP2 (endothelium of umbilical vein)	TIMP2 (blood vessel wall)	9	0.718	0.029
TIMP2 (endothelium of umbilical vein)	VEGF (endothelium of umbilical arteries)	9	0.716	0.030
TIMP2 (endothelium of umbilical vein)	VEGF (endothelium of umbilical vein)	9	0.775	0.014
TIMP2 (blood vessel wall)	TIMP2 (amniotic epithelium)	9	0.708	0.050
TIMP2 (extraembryonic mesenchyme)	HBD2 (amniotic epithelium)	8	0.728	0.041
TIMP2 (extraembryonic mesenchyme)	VEGF (endothelium of umbilical vein)	9	0.722	0.028
TIMP2 (extraembryonic mesenchyme)	VEGF (extraembryonic mesenchyme)	9	0.792	0.011
VEGF (endothelium of umbilical arteries)	HBD2 (amniotic epithelium)	8	0.733	0.038
VEGF (endothelium of umbilical arteries)	VEGF (blood vessel wall)	9	0.832	0.005
VEGF (endothelium of umbilical arteries)	VEGF (extraembryonic mesenchyme)	9	0.765	0.016
VEGF (endothelium of umbilical vein)	HBD2 (amniotic epithelium)	8	0.719	0.044
VEGF (endothelium of umbilical vein)	VEGF (blood vessel wall)	9	0.717	0.030
VEGF (endothelium of umbilical vein)	VEGF (extraembryonic mesenchyme)	9	0.843	0.004
VEGF (blood vessel wall)	HBD2 (amniotic epithelium)	8	0.846	0.008
VEGF (blood vessel wall)	VEGF (extraembryonic mesenchyme)	9	0.800	0.010
VEGF (blood vessel wall)	VEGF (amniotic epithelium)	9	0.861	0.003
VEGF (extraembryonic mesenchyme)	HBD2 (amniotic epithelium)	8	0.893	0.003
VEGF (extraembryonic mesenchyme)	VEGF (amniotic epithelium)	9	0.705	0.034

Abbreviations: *n*—pairs analysed in Spearman’s rank-order correlation analysis; r_s_—correlation coefficient (Spearman’s rho); *p* value—*p* values < 0.05 are considered statistically significant; MMP2—matrix metalloproteinase-2; TIMP2—tissue inhibitor of metalloproteinases-2; VEGF—vascular endothelial growth factor; HBD2—human beta defensin-2.

## Data Availability

The data presented in this study are available upon request from the corresponding author.
